# Casting a wide net: use of diverse model organisms to advance toxicology

**DOI:** 10.1242/dmm.043844

**Published:** 2020-04-01

**Authors:** Mark E. Hahn, Kirsten C. Sadler

**Affiliations:** 1 Biology Department, Woods Hole Oceanographic Institution, 45 Water Street, Woods Hole, MA 02543, USA; 2 Boston University Superfund Research Program, Boston, MA 02118, USA; 3 Program in Biology, NYU Abu Dhabi, PO Box 129188 Abu Dhabi, UAE

**Keywords:** Animal models, Toxicants, Toxicology, Zebrafish

## Abstract

Toxicology – the study of how chemicals interact with biological systems – has clear relevance to human health and disease. Persistent exposure to natural and synthetic chemicals is an unavoidable part of living on our planet; yet, we understand very little about the effects of exposure to the vast majority of chemicals. While epidemiological studies can provide strong statistical inference linking chemical exposure to disease, research in model systems is essential to elucidate the mechanisms of action and to predict outcomes. Most research in toxicology utilizes a handful of mammalian models that represent a few distinct branches of the evolutionary tree. This narrow focus constrains the understanding of chemical-induced disease processes and systems that have evolved in response to exposures. We advocate for casting a wider net in environmental toxicology research to utilize diverse model systems, including zebrafish, and perform more mechanistic studies of cellular responses to chemical exposures to shift the perception of toxicology as an applied science to that of a basic science. This more-inclusive perspective will enrich the field and should remain central to research on chemical-induced disease.

## Abstract (precis)

**Summary:** This Editorial highlights the value of utilizing evolutionarily diverse model systems, including zebrafish, in basic toxicology research to investigate mechanisms of chemical-induced disease.

## Introduction

In the landmark book *Silent Spring*, the celebrated marine biologist, environmental activist and writer Rachel Carson boldly stated that “For the first time in the history of the world, every human is being subjected to contact with dangerous chemicals, from the moment of conception until death” (Carson, 1962)*.* This rings true today as, in the nearly 60 years since this book was published, chemicals synthesized or unearthed by humans have been flooding our environment at an unprecedented rate. The news is replete with stories of people suffering from unintentional exposure to metals, industrial chemicals, pesticides and other toxicants. While the effects of acute exposure gain significant clinical and media attention, we now know that even transient exposure to chemicals can have dire, long-lasting consequences, including reproductive disorders, metabolic disease, neurologic disorders and cancer. Chemicals in the environment affect not only humans, but also fish, wildlife, plants and microorganisms. These interactions drive evolution, as organisms adapt to persistent exposures, and, most relevant to readers of Disease Models & Mechanisms, can cause devastating diseases. Thus, more than ever, the spotlight is on toxicologists to uncover the basic mechanisms of how organisms interact with their chemical environment and to forecast the impact of these interactions on health now and in the future.

## Contact with chemicals: impacts on human and animal health

All animals, including humans, interact with chemicals in food, air and water. Aquatic animals are immersed in a chemical solvent, as the lakes, streams and oceans that are home to most of the life on our planet are full of natural products – including metals, metalloids and organics – and a dizzying array of synthetic chemicals. Exposure to many of these is unavoidable. The cellular and genetic mechanisms that respond to these chemicals are conserved across species, as evolution has selected for robust and integrated defense mechanisms to mitigate the stress and damage that chemicals can inflict on cells and physiological systems.

Both natural and synthetic chemicals can interfere with biological systems, and cells have evolved distinct pathways to manage their toxic effects, including metabolic enzymes that alter harmful chemicals to reduce their damaging effects, antioxidants that protect against the reactive oxygen species that many toxicants induce, and DNA repair mechanisms to undo some of the damage caused by agents that interact with the genome. Natural toxicants include elements such as arsenic, lead and mercury that are part of the Earth's crust; these leach into water, are aerosolized in dust, and can become incorporated into the cells of plants and animals that form the base of the food chain. In addition, many organisms produce a wide array of organic natural compounds, including potent toxicants that can be found in the food we eat. Synthetic chemicals produced by humans have reached the farthest corners of the Earth. The pervasive use of plastics, industrial solvents, pesticides and many other organic chemicals, combined with inefficient waste management systems, have spread synthetic chemicals to remote areas: fish inhabiting the deepest areas of the ocean are contaminated with industrial pollutants (Jamieson et al., 2017; Stegeman et al., 1986) and even humans inhabiting remote regions of the world may be affected by toxicant exposures (Donaldson et al., 2010). Such toxicants in the environment can drive evolution, as illustrated by studies of fish populations that have adapted to polychlorinated biphenyl (PCB) pollution through selection for gene variants that desensitize a pathway involving the receptor that binds to these organic toxicants (Wirgin et al., 2011). Therefore, toxicant exposure can cause acute effects resulting in disease or lethality as well as provide evolutionary pressure to change the genetic equilibrium of a population.

## Model organisms in toxicology: casting a wide net

Studies to identify the molecular mechanism underlying disease-related outcomes of toxicant exposure have traditionally relied on the use of mammalian model organisms or human cells in culture. These serve as a proxy for understanding how humans respond to such exposures. However, lessons from evolution that reveal how organisms have adapted to chemicals are among the most powerful in biology. Since mammals are not unique in their response to chemicals, we argue for an expansion of the model organism pool used to study toxicology.

The recent declaration by the US Environmental Protection Agency (EPA) that the use of mammals for chemical testing will be phased out (https://www.epa.gov/newsreleases/administrator-wheeler-signs-memo-reduce-animal-testing-awards-425-million-advance) echoes earlier calls by the EPA and the National Institutes of Health (NIH) to move away from *in vivo* studies in mammalian models (Collins et al., 2008). These calls require a renewed search for alternatives. Indeed, the EPA mandate demands it.

The ability to carry out well-controlled experiments in established models is essential for the rigorous evaluation required of toxicological studies. We can learn even more from well-executed studies in a wide range of biological systems (Duronio et al., 2017; Howe et al., 2018). Research to understand how diverse animals interact with a chemical environment ultimately advances human health as it deepens our perspectives on adaptation, evolution and the function of cellular stress responses (Ballatori and Villalobos, 2002).

In addition to including a more diverse range of model organisms in toxicology, we advocate for changing the classical perception of the field. Toxicology is more than applied science, focused solely on toxicity testing; it is indeed an experimental science, firmly rooted in fundamental cell, molecular and developmental biology, and focused on the molecular mechanisms by which chemicals interact with cells and physiological systems. Toxicologists, biochemists, cell biologists and translational scientists have discovered the integral role played by stress signaling pathways in response to toxicants, and thus studies in environmental toxicology serve to bridge across disciplines. Reproductive health, development, cancer, metabolism, neurocognitive function, aging, immunity and our symbiotic microbiome are all affected by exposures to toxicants, and studying disease mechanisms therefore relies on rigorous basic science in the field of toxicology. We entreat our colleagues invested in disease-related research to consider the mechanistic research in toxicology as basic research, as these studies advance our fundamental understanding of ligand-activated transcription factors, biotransformation enzymes and other systems that interact with toxicants in the cell (Hahn, 2019). By broadening our perspective, we will learn from the fundamental findings of toxicology studies in systems across the evolutionary spectrum, providing more comprehensive insights into how organisms adapt or fail in our chemical-laden environment.

## Zebrafish as a model and tool for toxicology research

Among the many non-mammalian model systems that can contribute to fundamental understanding in toxicology, zebrafish is one for which potential is being fully realized, with hundreds of research groups worldwide using this system to study toxicology. Zebrafish are well established for studying vertebrate development and as a translational model in disease research (Patton and Tobin, 2019). More recently, zebrafish have risen to the fore as the only vertebrate model amenable to high-throughput screening as well as focused mechanistic studies to identify the cellular and genetic factors that promote disease in response to toxicants (Bambino and Chu, 2017; Horzmann and Freeman, 2018). Our own laboratories utilize zebrafish and other traditional and non-traditional model organisms because asking similar questions across a range of systems provides richer and more meaningful insight.

Our choice of zebrafish is motivated by the widely available and well-characterized tools and reagents amenable for use in this system, the ability to perform manipulative experiments using both genetic and pharmacological approaches, a well-annotated genome, an engaged and collegial community of researchers, and a wealth of knowledge about their cell biology, physiology and reproduction. The long history of toxicological research in rodents can then serve as a basis of comparison for findings in zebrafish and other organisms so that we can identify those responses to toxicants that are shared across species and those that have evolved in a species-specific fashion.

Zebrafish embryos develop rapidly in a Petri dish, making exposure to most compounds as simple as addition to their water, allowing for full control of exposure windows, determination of dose-response relationships, and assessment of a variety of disease-related outcomes. These features facilitate high-throughput screening, which is being used in both drug development and toxicity testing (Wiley et al., 2017). Moreover, there is high conservation in the genes and pathways that mediate the response to chemicals: the liver is well developed by 5 days post-fertilization and is responsible for xenobiotic metabolism mediated by enzymes including the cytochrome P450 system (Goldstone et al., 2010). The cellular responses to stress are well conserved between zebrafish and mammals, including systems that protect against oxidative stress and DNA damage (Fuse and Kobayashi, 2017; Hahn et al., 2015). Zebrafish research has uncovered mechanisms of toxicity, and toxicant modifiers of disease susceptibility for many environmental toxicants, including naturally occurring elements like arsenic (Bambino and Chu, 2017) and anthropogenic pollutants such as dioxin (Carney et al., 2006; King-Heiden et al., 2012). The power of this system is also being used for whole-organism histological phenotyping (Ding et al., 2019) and for understanding how genetic variation influences the response to toxicants (Balik-Meisner et al., 2018).

We echo colleagues who have proposed a distinction between a ‘model’, in which there is an accurate representation of the phenotype being investigated, and a ‘tool’, which provides mechanistic insight without necessarily fully recapitulating the disease phenotype (Sive, 2011). We view zebrafish both as a model for studying the effects of toxicants that have a universal effect on eukaryotic cells, such as DNA-damaging agents or metals, and as a tool for investigating processes, such as real-time evaluation of developmental mechanisms, that are challenging to study in mammals.

## Natural and synthetic toxicants: arsenic and dioxin as examples

### Arsenic

Metals and metalloids are widespread toxicants that have historically been perceived as the most dangerous, as they are ubiquitous elements in the Earth's crust. Arsenic seeps into the groundwater used for drinking and irrigation and is mobilized in dust storms. Arsenical pesticides have been used widely to control pests in crops and as an agent of warfare, and as such, “arsenic provides a classic case of the virtually permanent poisoning of the soil” (Carson, 1962). Arsenic is listed by the US Agency for Toxic Substances and Disease Registry (ATSDR) as the #1 chemical contaminant due to its substantial contribution to disease and the extensive risk to people. The impact of chronic arsenic exposure on human health is profound – skin lesions, cancer, diabetes and other metabolic diseases are disproportionally high in regions with chronic arsenic exposure. Acute arsenic poisoning can be fatal. The World Health Organization (WHO) estimates that 200 million people worldwide are exposed to arsenic levels exceeding the recommended limit (10 µg/ml). Of these, between 10-30% of those at risk live in Bangladesh and West Bengal (India), regions described as the site of the largest mass poisoning in history. This massive public health issue makes understanding the mechanisms of arsenic toxicity an urgent and unmet need.

To understand how arsenic affects cell biology *in vivo* and to predict long-term health outcomes, researchers have traditionally used mice and rats as these are assumed to approximate human physiology. However, rodents do not fully recapitulate the carcinogenesis, metabolic disease and skin disorders found in exposed humans (States et al., 2011). Since arsenic is present in rivers, streams, lakes and oceans, animals in these environments are also affected. Many groups have used zebrafish to investigate the metabolic, behavioral and developmental effects of arsenic. In our own work, we have focused on the observation that human populations exposed to arsenic have a significantly increased risk of liver disease. We recapitulated this in zebrafish by showing that arsenic causes lipid accumulation and a robust oxidative stress response in the liver, and that subtoxic exposure to both arsenic and alcohol synergizes to enhance the oxidative stress response, leading to endoplasmic reticulum (ER) stress and fatty liver (Bambino et al., 2018). We conclude that arsenic and alcohol share intracellular targets, including those involved in generating oxidative stress (Fuse et al., 2016), which makes the ER dysfunctional. These findings highlight how zebrafish provide a novel platform to investigate toxicant interactions and to uncover the mechanisms of disease-relevant outcomes of exposures.

### Dioxin

Carson (1962) observed that “the chemicals to which life is asked to make its adjustment are no longer merely the calcium and silica and copper and all the rest of the minerals washed out of the rocks and carried in rivers to the sea; they are the synthetic creations of man's inventive mind…having no counterparts in nature”. The so-called dioxin-like chemicals (DLCs) – halogenated aromatic hydrocarbons that resemble polychlorinated dibenzo-*p*-dioxins in their effects and mechanisms – provide one such example. Although naturally occurring in trace amounts, DLCs are primarily of anthropogenic origin and are now widely distributed in environments worldwide as a result of improper disposal and environmental transport. The health consequences of DLC exposure can be severe, including cancer, developmental defects and cardiovascular disease, and can have devastating effects in wildlife (Cook et al., 2003) and humans (Eskenazi et al., 2018). These devastating risks make DLCs among the most widely studied toxicants.

Although much has been learned about the toxicity of DLCs and their mechanism of action from studies in mammals (Pohjanvirta and Tuomisto, 1994), research using zebrafish has provided novel insights into mechanisms involved in their developmental toxicity. For example, a series of elegant studies in zebrafish embryos exposed to the most toxic DLC, 2,3,7,8-tetrachlorodibenzo-*p*-dioxin (TCDD), revealed the cellular and molecular mechanisms underlying the effects on cardiovascular development. TCDD was shown to prevent formation of the epicardium and downregulate *sox9b*, an important gene in heart development (Hofsteen et al., 2013; Plavicki et al., 2013). Additional studies demonstrated that craniofacial malformations in TCDD-exposed zebrafish embryos also resulted from downregulation of *sox9b* (Xiong et al., 2008), mediated by the induction of an inhibitory long non-coding RNA (Garcia et al., 2018). TCDD also inhibits tissue regeneration, and studies in zebrafish showed that the mechanism involves altered signaling through the Wnt pathway (Mathew et al., 2008). These examples demonstrate how zebrafish research advanced our understanding of the mechanisms of DLC toxicity, complementing and extending discoveries made using mammalian models.

## Food for thought on the future of diverse animal models in toxicology research

Human cells in culture and rodents have been the standard laboratory models for studying the ability of toxicants to cause disease in humans. Understanding chemical-induced disease in humans is an important goal, and the urgency of such work cannot be overstated. However, studies focused only on mammalian systems provide a limited perspective on how toxicants affect cell biology. The recent EPA declaration forgoing the use of mammals in future chemical testing efforts frequently interchanges the terms ‘animal’, ‘vertebrate’ and ‘mammal’, reflecting a narrow biological perspective that equates all animals with vertebrates and all vertebrates with mammals. We have experienced similar erroneous assumptions in discussions with colleagues. Scientists wedded to toxicology research using rodents frequently express surprise upon learning that zebrafish biology is closely related to that of mammals, and that fish, like their drier vertebrate relatives, have livers that metabolize chemicals, get cancer and possess the same stress response pathways. This highlights the perceptions of some researchers and policy makers that only mammalian models can yield data with relevance to human health. However, with the pronounced changes in the EPA and NIH positions on the use of mammals in chemical testing, many researchers will be driven to explore other animal systems. The tree of life is vast and animals have evolved ways to manage and even thrive in their chemical environments. As Carson encouraged nearly 60 years ago, we can learn from studying the natural world, with “new, imaginative and creative approaches” to advance toxicology.
